# Palliative care management by caregivers in home care: theoretical validation in a conversation circle

**DOI:** 10.1590/0034-7167-2021-0737

**Published:** 2022-09-09

**Authors:** Roberta Teixeira Prado, Denise Rocha Raimundo Leone, Thiago de Medeiros Souza, Paula Valente Werneck, Maria Ribeiro Lacerda, Edna Aparecida Barbosa de Castro

**Affiliations:** I Faculdade de Ciências Médicas e da Saúde de Juiz de Fora. Juiz de Fora, Minas Gerais, Brazil; II Universidade Federal de Juiz de Fora. Juiz de Fora, Minas Gerais, Brazil; III Universidade Federal do Paraná. Curitiba, Paraná, Brazil

**Keywords:** Validation Study, Grounded Theory, Palliative Care, Caregivers, Home Care, Estudio de Validación, Teoría Fundamentada, Cuidados Paliativos, Cuidadores, Asistencia Domiciliaria, Estudo de Validação, Teoria Fundamentada, Cuidados Paliativos, Cuidadores, Assistência Domiciliar

## Abstract

**Objectives:**

to present the validation process of a Grounded Theory on the management of palliative care at home by a caregiver of a family member who experiences a death/dying process.

**Methods:**

a qualitative, explanatory research, which validated a theoretical matrix through a conversation circle containing 15 family caregivers and nine healthcare professionals, in December 2018.

**Results:**

forty-six propositions were validated regarding family caregivers’ contextual, causal, intervening conditions, consequences and action strategies to deal with the dying and death process of a family member. Conversation circles encouraged dialogue and (re)signification of the senses and knowledge of those involved, proving to be a way of educating and promoting the exercise of citizenship by participants.

**Final Considerations:**

the conversation circle made it possible for participants to interact and share information and experiences regarding home care for palliative patients and their families.

## RESUMO

**Objetivos:**

apresentar o processo de validação de uma Teoria Fundamenta nos Dados sobre a gestão do cuidado paliativo domiciliar pelo cuidador de um familiar que vivencia processo de morte/morrer.

**Métodos:**

pesquisa do tipo explicativa, qualitativa, que validou uma matriz teórica por meio de uma roda de conversa contendo 15 cuidadores familiares e nove profissionais de saúde, em dezembro de 2018.

**Resultados:**

foram validadas 46 proposições relativas a condições contextuais, causais, intervenientes, consequências e estratégias de ação de cuidadores familiares para lidar com o processo de morrer e morte do familiar. A roda de conversa impulsionou o diálogo e a (re)significação dos sentidos e dos saberes dos envolvidos, demonstrando ser uma forma de educar e propiciar o exercício da cidadania dos participantes.

**Considerações Finais:**

a roda de conversa possibilitou a interação dos participantes e o compartilhamento de informações e experiências diante do cuidado domiciliar de pacientes paliativos e seus familiares.

## RESUMEN

**Objetivos:**

presentar el proceso de validación de una Teoría Fundamentada en Datos sobre el manejo de los cuidados paliativos en el hogar por parte del cuidador de un familiar que vive un proceso de muerte/morir.

**Métodos:**

investigación cualitativa, explicativa, que validó una matriz teórica a través de un círculo de conversación con 15 cuidadores familiares y nueve profesionales de la salud, en diciembre de 2018.

**Resultados:**

se validaron 46 proposiciones sobre condiciones contextuales, causales, intervinientes, consecuencias y estrategias de acción de los cuidadores familiares para enfrentar el proceso de morir y la muerte del familiar. El círculo de conversación estimuló el diálogo y la (re)significación de los sentidos y saberes de los involucrados, demostrando ser una forma de educar y promover el ejercicio de la ciudadanía por parte de los participantes.

**Consideraciones Finales:**

el círculo de conversación posibilitó que los participantes interactuaran y compartieran información y experiencias sobre el cuidado domiciliario de los pacientes paliativos y sus familias.

## INTRODUCTION

With the recent interlocutions between palliative care (PC) and home care (HC), the home has become a place of assistance support for people who live the end of life^(1)^. In this, continuity of care is established for people with chronic diseases, dependent on care that is not always available in a hospitalization^(2)^. The home is an important place of care, as it allows the approximation between care interventions and the life context of people with PC and their families, considering their biopsychosocial and spiritual needs^(3)^.

However, demands have been expressed to the family, which, with its own efforts and costs, takes care of one of its members, in most cases, alone, also requiring assistance and health support^(4)^. In the home context, in addition to the financial cost, transferred to users and their family, there is also the emotional and social costs. Among other situations, the emotional costs are represented by the feelings involved in caring and the wear and tear of living with a family member with a disease without therapeutic possibility. The social cost is expressed by isolation, by abandoning leisure activities, in short, by “cloistering” within their own residence, when dedicating themselves almost exclusively to caring for the other^(5)^.

A large review study on the effectiveness and costs of home PC for adults with advanced diseases and their caregivers has provided clear and reliable evidence that they alleviate patients’ symptoms, improve the chance of choosing to die at home without grief modification. caregivers, thus justifying the provision of this type of care. However, the evidence on the effects of home PC is inconclusive, from the perspective of caregivers, and how home PC is managed for the family. The rapid aging of populations with greater complexity and need for home PC is an international challenge, and existing studies are based on health services and systems in developed countries, justifying studies in middle and low-income countries when a family member is supported by home PC care models^(6)^.

In Brazil, the macro care context in which the intertwining between family and health services is disseminated by HC^(1)^ lacks studies that theorize how caregivers understand and experience the PC of their family members in a shared way with healthcare professionals, i.e., how the family has managed the death/dying process of a family member at home since the implementation of this policy.

With these questions, and due to the aforementioned research gaps on this phenomenon^(6)^, we developed a substantive theory with the Straussian perspective of Grounded Theory (GT), an explanatory, inductive-deductive qualitative research method, focused on human action-interaction^(7-11)^, which proposes to develop concepts and explanations on phenomenon delimited to specific context^(12-13)^.

In the national and international scientific literature on GT, there are few studies that specifically discuss the step of validation of substantive theory^(10,14-16)^. Even more limited are studies that used the conversation circle (CC), which was the validation technique^(14)^ chosen by us. Moreover, the studies we identified address phenomena related to the experiential process of a professor, who is a physician, on the professional training of medical students^(16)^, interactive processes in the support network for individuals with tuberculosis^(15)^ and in-service training model on the Nursing Process from the point of view of teaching-service integration^(14)^. We did not identify theoretical validations on HC management by family members who experience the dying and death process, considering macro-sociological contexts of contemporary public health in which discharge for continuity of HC services require the presence of a family caregiver^(1)^.

We understand that evidence based on knowledge, experiences and experiences of members of a social group who experience the phenomenon are essential to support the actions of healthcare professionals and programs/public policy to support a family/family caregiver in the process of discharge for PC through HC.

## OBJECTIVES

To present the validation process of a GT on the management of PC at home by a caregiver of a family member who experiences a death/dying process.

## METHODS

### Ethical aspects

The research complied with the ethical precepts of Resolution 466/2012 of the Brazilian National Health Council, and was submitted to the Research Ethics Committee of a public university. Codes were used for participants’ citations, with FCV for family caregiver validator and HPV for healthcare professionals validator, followed by the order number in the databases.

### Study design

This is qualitative, explanatory research guided by the Straussian current of GT^(12)^, presented according to the COnsolidated criteria for REporting Qualitative research (COREQ).

### Theoretical-methodological framework

To develop the theoretical matrix “Understanding home care management by the family in the face of the death/dying process”, we based ourselves on the conception of a paradigmatic model by Strauss and Corbin^(12)^, in the Brazilian National Home Care Policy (*Política Nacional de Atenção Domiciliar*)^(1)^ which, in order to shorten or avoid hospitalizations, control patients’ pain and suffering, proposes to offer PC in users’ home in dialogue with the family. We are also based on Cecílio’s multidimensional conception of care management, emphasizing, in this study, the family dimension, intrinsic to the world of life, where relationships can be conflicting, resulting from the complexity of family ties, the permanent requirements for carrying out care and the workload for caregivers^(17)^.

In GT, the collected data are analyzed comparatively and systematically^(11-12)^, and validation is a step that aims to check the rigor and reliability of the substantive theory produced^(12)^. It has been continuously reconstructed, identifying different methodological currents: the Glaserian or classic; the Straussian, relativist/subjectivist; the one guided by Kate Charmaz, constructivist; Adele Clark’s, which emphasizes situational cartographic analysis; and, more recently, the Corbinian-Straussian interpretation, resulting from a critical analysis of the first Straussian version, influenced by constructivism and contemporary postmodern thinking^(8,11,18)^.

From the Straussian perspective, validation constitutes an important stage of theoretical construction, which makes it possible to compare concepts and their relationships with the research raw data, understand participants’ reaction to the theoretical matrix appreciation, to determine how much they represent the investigated phenomenon, prevent or explain what happens within the substantive theory for such investigation and its applicability. The researcher refines the theory, removes excesses and completes the categories that may be poorly developed, hopes to obtain a conditional/consequential model of the investigated phenomenon, demonstrating the interaction of conditions/consequences and actions/interactions, in addition to the existing connectivity, denoting the data complexity and richness^(9-12,14-18)^.

The purpose of this article is to give visibility to the validation stage, as it contributes to the rigor and quality of research with a qualitative approach, aiming to enhance the accuracy and reliability of its results, minimizing risks of bias by researchers^(19)^. Data reliability and validity are necessary for refining scientific investigation and knowledge production, contributing to verify the representativeness of findings and assess the quality of a scientific study^(20)^. However, validity and reliability are common concepts in quantitative research and also objects of discussion in qualitative studies^(15)^.

In this approach, validation allows verifying whether the results and conclusions of a survey are valid, considering the sampling and the context in which the investigation was carried out^(21-22)^. To this end, specific strategies are used, such as verification of members, when the interpretation of the reports made by the researcher is compared with that of the research participants, establishing a degree of correspondence between them and obtaining an understanding and explanation of the phenomenon^(23)^.

Among the validation strategies by verification of members, CC is a technique previously used for theoretical validation, which encourages transformations and innovations in the context in which the phenomenon is expressed, as in professional practice, emphasizing GT contributions in the construction of substantive and formal theories^(15-16)^.

### Methodological procedures

For the validation process, CC was adopted anchored in Paulo Freire’s pedagogical proposal, as it allows the expression and construction of meanings from everyday and shared experiences^(24-25)^, encouraging the participation of people through conversation, encouraging reflection and enabling the understanding of reality^(26-29)^. The validation process did not depart from the methodological path, in which there was constant comparison, and participants were given the opportunity to correct, in a timely manner, any errors in the data, analyzes and interpretations, in order to guarantee report credibility^(30)^.

### Study setting

The research that originated this study was carried out in the participants’ natural environment (home or workplace), and CC, for data validation, took place in December 2018 in a public home care service (HCS) auditorium in Minas Gerais.

### Data source

The research developed in the post-doctoral stage, which generated a substantive theory, included, for convenience, 18 participants over 18 years old who had experience in HC for people in the death/dying process. Initially, nine family caregivers participated and then nine professionals, due to the phenomenon specificity and also because they were mentioned in caregivers’ reports.

CC for data validation was composed of 24 participants, 14 family caregivers, a caregiver hired by the family, eight professionals who are HCS members and one from the Primary Healthcare service.

The criteria established for the selection of validating professionals were being a healthcare professional and having professional experience with the terminality and death of patients in a home environment or being a home caregiver and accompanying a patient with a PC indication, in addition to having time available to participate in the research and sign the Informed Consent Form (ICF).

The invitation to CC contained the objectives, date and time, and was made by the HCS to all family caregivers of people with PC during consultations, and to professionals, by email. Three professionals and a family caregiver previously participated in the research. The absence of participating caregivers occurred due to death or not having someone to leave a family member with at the time of CC, and the absence of professionals, due to a portion having to maintain routine care and intercurrences.

### Data steps, collection and organization

Data collection, interpretation and analysis is concomitant in GT, enabling the construction of substantive theory supported by propositions based on the data obtained. According to the Straussian perspective, propositions are organized in the categories of a paradigmatic model that aims to explain the phenomenon, pointing out its context of occurrence, its causes, consequences, intervening conditions and action/interaction strategies^(12)^.

**Figure 1:**
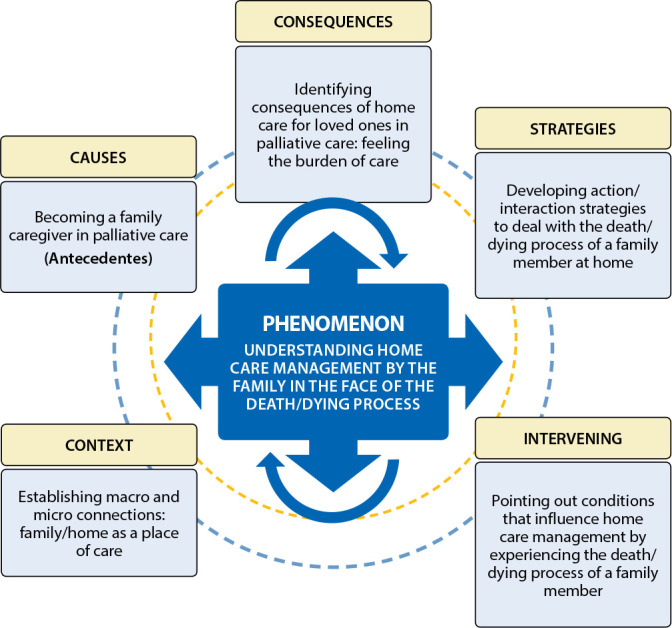
Substantive theory “Understanding home care management by the family in the face of the death/dying process”, Juiz de Fora, Minas Gerais, Brazil, 2019

After theoretical saturation and obtaining a theoretical scheme, we started the validation process, which took place in three stages. In the first one, we compared it with the raw data of this research, refining and delimiting the propositions, according to the categories, obtaining the theoretical matrix to be validated and its synthesis in a flowchart (Figure 1). In the second, the matrix was presented, analyzed, discussed, assessed and validated in CC, and in the third, we processed, analyzed and discussed in the group of researchers the data obtained in CC. This was a long and laborious phase, involving tabulation of results, transcription of recording and reading, observation notes, recoding and reordering the propositions, according to agreement and disagreement. In addition to these steps, we emphasize that validation began with the project construction and development process, safeguarding the auditability (discussions and consensus in a research group, with a supervisor and external referee to the group) and credibility criteria, safeguarding the methodological rigor, fidelity to the framework, triangulation with the CC method and with other research on the topic, in addition to being screened by an ethics committee.

The CC strategy was conducted by a doctor in post-doctoral internship, supported by an undergraduate nursing student. We have developed two instruments exclusively for this moment, containing the phenomenon properties, dimensions, structure and processes in the form of 46 propositions, extracted from incidents and codes, foundations of the categories that composed the paradigmatic model. One has 46 propositions, presented to family caregivers, and another, 35, presented to healthcare professionals, as we removed 11 propositions that were specific to the conditions reported by caregivers ahead of HC management. Both were in language accessible to each group. In Chart 1, we list the propositions submitted for validation, being six causal, 13 for consequences, 12 contextual, seven related to actions and strategies and nine to intervening conditions.

**Chart 1:** Propositions validated by family caregivers and healthcare professionals, Juiz de Fora, Minas Gerais, Brazil, 2019

Propositions according to categories of substantive theory	Answers^(^*^)^ FCV (n =15) HPV (n=9)
**Category 1 - Causal condition:** Becoming a family caregiver in home palliative care	
**(P1)** Between staying in the hospital and receiving care at home, the family preferred to take and keep at home a family member with chronic conditions and dependences, understanding that they would have more affection, care and less risk of complications. They recognize that this decision causes exhaustion, physical and emotional exhaustion in family caregivers.	FCV (15) HPV^(^**^)^
**(P2**) One learns to care for a family member from the beginning of dependence on care at home and, progressively, with daily experiences.	FCV (14) HPV^(^**^)^
**(P3**) Care is taken with love and care, recognizing that PC is temporary, due to the severity of a family member’s disease.	FCV (12) HPV^**^
**(P4**) Daily activities are organized according to family members’ health condition and care needs.	FCV (12) HPV^(^**^)^
**(P5**) They know that a family member is close to death, they anticipate that they will miss them, but they have the feeling of accomplishment, due to the effort to do the best they can.	FCV (11) HPV^(^**^)^
**(P6)** They dedicate themselves to caring for family members at home, leaving aside them own care and needs.	FCV (9) HPV^(^**^)^
**Category 2 - Consequences:** Identifying consequences of home care for loved ones in palliative care: feeling the burden of care	
**(P7)** Caring for the loved one in PC was considered a life lesson, experience formation and possibility of maturation.	FCV(15) HPV (5)
**(P8)** Taking on HC of a family member with PC brought about several changes in caregivers’ lives.	FCV (12) HPV (9)
**(P9)** The maintenance and control of domestic activities, financial administration and acquisition of materials and medicines were accumulated with the various functions and responsibilities of direct care of a family member, such as bathing, hygiene, food and other primary care.	FCV (10) HPV(9)
**(P10)** They started to sleep less than they slept before taking on care.	FCV(9) HPV^(^**^)^
**(P11)** Financial difficulties increased due to expenses with family care under PC.	FCV(9) HPV(9)
**(P12)** They have a feeling that “life stopped” after taking care of a family member. They felt that they were living a family member’s life more than theirs.	FCV (9)^*^ HPV^**^
**(P13)** Many times, a caregiver has doubts, uncertainties, feels stressed, with fear, anguish and sadness.	FCV (9) HPV(9)
**(P14)** They dedicate more time to care activities, and this hinders going out to work or performing some paid activity.	FCV(9) HPV(9)
**(P15)** They feel physical, emotional, social and financial burden, for having to take over a family member’s home PC.	FCV (8) HPV(8)
**(P16)** A caregiver needs more care than a patient in HC because they are worn out.	FCV (8) HPV(9)
**(P17)** Relationships with other family members and friends were impaired, and interaction with other people was reduced.	FCV(8) HPV(9)
**(P18)** They almost never left the house, and the opportunities to go out were reduced.	FCV(7) HPV(9)
**(P19)** The psychological condition from before has changed: sometimes they feel angry, other times they are quieter, sadder and cry.	FCV (6) HPV(9)
**Category 3: Strategies and actions**: Developing action/interaction strategies to deal with the death/dying process of a family member at home	
**(P20)** We seek to provide a family member what they need the most: love and the presence of the family.	FCV (14) HPV(9)
**(P21)** Life experiences, religion/belief/faith, clarification and support received from professionals, friends or family members help to deal with a family member’s condition of dependence.	FCV (13) HPV (8)
**(P22)** They recognized as support for HC management the receipt of materials needed for dressings, hygiene and others, according to a family member’s care needs and availability at the HCS.	FCV (12) HPV (9)
**(P23)** They recognized that a necessary action is to assimilate the terminality of the loved one’s life, but it is usually difficult to think about the loss, despite knowing its severity.	FCV (11) HPV^(^**^)^
**(P24)** Previous experiences in caring for another sick person contributed to alleviate the way of reacting in the care of a family member with a disease that threatens continuity of life and that has PC indication.	FCV (10) HPV(7)
**(P25)** Each person reacts differently to a disease that threatens continuity of life and is indicated for undergoing PC treatment.	FCV (10) HPV(7)
**(P26)** Over time, caring for a family member in PC at home facilitated the acceptance of end of life, by closely monitoring the reactions to the disease, treatment and understanding how the disease threatens continuity of life.	FCV (9) HPV^(^**^)^
**Category 4: Contextual conditions:** Establishing macro and micro connections: family/household as place of care	
**(P27)** Not all families know the day when the HCS team will make a home visit, unless a procedure is scheduled, such as material collection for exams.	FCV (13) HPV(7)
**(P28)** The HCS is a reference for HC.	FCV (12) HPV(8)
**(P29)** Visits by team healthcare professionals occur whenever possible, weekly, varying according to the types of patients’ needs and possible aggravations or complications.	FCV (12) HPV(9)
**(P30)** Professionals should identify weaknesses in family caregivers, seeking to help them, since the experience of a family member with an incurable, progressive disease that threatens continuity of life changes the house routine, and family members also fall ill.	FCV (11) HPV(8)
**(P31)** A difficulty for a caregiver and family is to talk about the death/dying process experienced by a family member in home PC.	FCV (10)^*^ HPV^**^
**(P32)** The family takes over a large part of the costs generated by HC, especially with materials and health services that they cannot obtain through the Unified Health System (SUS - *Sistema Único de Saúde*). This tightens the family budget even more, as wages are low and spending is high. Sometimes it is necessary to make “sacrifices” to meet, in the best possible way, a family member’s care needs.	FCV (10) HPV(6)
**(P33)** Family caregivers need emotional and social support, and not only help to provide care and alleviate financial difficulties.	FCV (8) HPV(8)
**(P34)** Rarely, the team’s health workers talk about PC.	FCV (7) HPV(2)
**(P35)** Caregivers are not always supported by other family members.	FCV (6) HPV(8)
**(P36)** Rarely, someone among healthcare professionals talks about end of life and the possibility of a family member’s death.	FCV(7) HPV(1)
**(P37)** When healthcare professionals talk to a caregiver about the death/dying process with family members, they usually do not talk openly.	FCV(7) HPV (1)
**(P38)** When it happens that a healthcare professional talks about the death and dying process, who approaches me more directly is a doctor or psychologist.	FCV (6) HPV (5)
**Category 5: Intervening conditions:** Conditions/actions/interaction that modify the course of occurrence of the phenomenon	
**(P39)** Both nurses and nursing technicians provide guidance and care to family members when they pay a visit.	FCV (14) HPV(8)
**(P40)** There are doubts and difficulties in carrying out the procedures, but when they ask for help from HCS professionals, they are promptly answered. Professionals visit on the same day or the next day.	FCV (13) HPV (9)
**(P41)** The HCS team seeks to offer comfort and quality of life to patients under PC, extending emotional support to the family whenever possible.	FCV (13) HPV (9)
**(P42)** Communication between professionals and caregivers is important in HC. Family members need to feel comfortable asking questions and talking about their feelings and needs.	FCV (13) HPV (9)
**(P43)** The treatment offered by HCS professionals goes beyond clinical and technical aspects of care, as they often pay attention, provide guidance and words of comfort.	FCV (12) HPV (8)
**(P44)** Based on professionals’ guidance that a family member’s health status is advanced, caregivers communicate with HCS and request assistance whenever they need it.	FCV (12) HPV (9)
**(P45)** The support network of family caregivers is quite variable, including different sources, such as HCS, family members, neighbors, religion/spirituality/faith, among others.	FCV (10) HPV (7)
**(P46)** The more frequent presence of psychologists in the home visit would help families to talk about the death/dying process and, consequently, to deal with it better.	FCV (9) HPV (9)

FCV - family caregiver validator; HPV - healthcare professionals validator; HCS - Home Care Service; HC - home care; PC - palliative care; ^(^*^)^ Considering the answers to criterion 1 (always happens); ^(^**^)^ *Proposal not included in the HPV instrument.*

We printed copies of the instruments that were delivered during a CC, in order to trigger the participatory discussion, to work reflexively on contributions from participants, essential to the proposed validation process. The discussion about the propositions evolved in the form of an open press conference, which was recorded with prior authorization. Family caregiver validators (FCV) wrote a number next to each proposition, considering one of three possibilities: 1 - “That’s right! It fully corresponds to my reality”; 2 - “Not always! Only sometimes does it correspond to my reality”; 3 - “It does not correspond to my reality”. In the same way, healthcare professionals validators (HPV) validators, considering: 1 - “Yes, this corresponds to what happens or what I observe or live in my work with these families”; 2 - “Not always! Only sometimes”; 3 - “This does not correspond to what happens or what I observe or experience in my work with these families”.

We highlighted five moments during a CC, which was the second stage of validation. They are: 1) with participants arranged in a circle, we elucidate the purpose of the meeting, the validation process, its relevance, how it would occur, requesting authorization to record the conversation with a smartphone and applying the ICF; 2) gave a brief oral presentation of the research categories, explaining the flowchart (Figure 1) in accessible language using a PowerPoint presentation; 3) with the agreement of those present, the instruments containing the propositions to be validated were distributed, giving a brief time to appreciate them. There was a manifestation of doubts and clarifications; 4) key moment in this validation process, because, in 40 minutes, with problematization techniques, group communication, we asked participants to express themselves about the propositions. It was a dynamic moment, with frank group discussion and emanation of individual opinions relevant to the validation, verbally and in writing in the space of the instruments; 5) closing, acknowledgments, collection of instruments and assessment of the validation process experienced, with suggestions and opinions.

### Data analysis

The data analysis process had the same rigor of analysis required in GT, and occurred through coding. The analysis occurred concomitantly with data collection, being conducted through three types of coding: open; axial; and selective. OpenLogos^®^, version 2.0, was used to perform textual editing of empirical data and open coding.

There are four central criteria for theoretical validation by GT to judge the applicability of substantive theory to the studied phenomenon: adjustment; understanding; theoretical generalization; and control^(11)^. In our analysis, we confronted the substantive theory, respectively, with the first three analyzing: 1) if the theoretical matrix, it was faithfully adjusted to their daily reality and to the studied context, not according to a statistical logic, but to the expressiveness of verbal responses associated with the written ones in the instrument; 2) if, in addition to being understandable for us, researchers, it was also understandable for participants, healthcare professionals, and it made sense for them through their verbal and non-verbal responses about the propositions at the time of discussion in CC; 3) if the theoretical matrix were abstract enough, including enough variation to make it applicable to a variety of HC home PC contexts, considering participants’ verbal, non-verbal and written responses; 4) we did not apply the theoretical matrix to other reality(ies) of PC through HC, since, in order to reach this criterion, another study is needed, in order to test whether the hypotheses that propose relationships between concepts can be used to guide further action and direct interventions.

## RESULTS

The theoretical matrix’s categories and subcategories were representative of the moments that participants used to live or lived during PC either as a family member or as a HC professional. Of the 46 propositions that supported the substantive theory, we consider that seven were not fully validated, given the partial disagreements between FCV in four (P17, P18, P19, P36) and between HPV in three (P7, P34, P37), and one (P38) was not validated by the total disagreement by both groups, requiring caution in transferability/application in other contexts and attention by other studies (Chart 1).

Considering the “understanding” criterion, participants considered that the theoretical matrix was representative and understandable, due to the immediate understanding and acceptance expressed in verbal and non-verbal manifestations at the time of discussion of the propositions in CC.

*What you said is very true, because when my mother-in-law went had dementia everyone felt bad.* (FCV1)

*I agree with everything. Everything has to do with me.* (FCV 2)

*I think this topic should be worked more with healthcare professionals, so that each profession understands the role and conduct of other professionals.* (HPV 3)

*I, as a professional and as a person, feel unprepared to talk about ‘palliativeness’ with family members and recognize the need to reflect on this subject and would like to seek this knowledge/reflection.* (HPV 5)

After the contextual insertion of the 24 validating participants, there was agreement, at the end of CC, that the theoretical matrix would reach abstraction capacity, being able to explain the phenomenon and having the potential to be transferred and applied in different HCS contexts. We observed that propositions’ contents were reported even before they were read during the substantive theory presentation and, also, affirmations by facial and body expressions were noted when reading each proposition. All were considered understandable, the answers were immediate, and there was agreement that the theoretical matrix, categories and subcategories represented the phenomenon revealing its complexity. In addition to the reports seized during CC, the records of occurrence/recurrence of propositions in the daily lives of these participants, as validators, was what consolidated, making the substantive theory validated^(12)^.

## DISCUSSION

CC allowed validating substantive theory “Understanding home care management by the family in the face of the death/dying process”, developed with GT, considering the criteria for adjustment, understanding and theoretical generalization. The environment generated by this strategy made it possible to understand concepts and opinions, enhance leading role and empowerment of validators through dialogue and autonomy, essential for building collective and contextualized knowledge about the theme with sharing, listening and speaking exercise^(24,28-29)^.

The validation process showed that the five categories constructed grouped propositions that are routinely repeated in family caregivers’ lives and in healthcare professionals’ work, some of which were more expressive when discussed in a CC. The disagreements focused on propositions that did not occur entirely, but sometimes, or did not occur according to the reality of life or work of some participants. Among the HPV, they were related to training and job tenure in the multidisciplinary team, and among the FCV, to singularities inherent to each one, associated with experiences, dedication time to the family and bond with the HCS.

Category 1 included six antecedents that justify the phenomenon and consolidate the existence of a family caregiver managing home PC. For the implementation of PC through HC, the decision and agreement of patients and family were evidenced as fundamental, and a family member needs to be willing to legally take over the role of caregiver and learn about care. The function is not instituted immediately, and one becomes a caregiver, constituting an experience and coming to understand it as a transitory condition due to the possibility/proximity of a family member’s death.

The process of becoming a caregiver is related to the degree of involvement between a caregiver and a person to be cared for, which is sometimes painful when the act of caring becomes an obligation or in cases of inexistence of an affective relationship between a caregiver and a person to be cared for. Moreover, becoming a caregiver is associated with a cognitive dimension, as it is necessary that they learn the health activities to be performed and that will become part of the daily care at home^(31)^.

Category 2 included 13 conditions resulting from the choice to care for a family member under PC at home, signaling the consequences of this decision for loved ones, such as burden. The maintenance and control of household activities, financial administration and the acquisition of materials and medicines necessary to meet the various functions and responsibilities of direct family care, such as bathing, hygiene, food and other basic care, are accumulated. The consequences seem mitigated for six caregivers considering the responses of ten of the propositions (P10 to P19) as being “sometimes”. Among other factors, the dialogue with the HCS and the presence of professionals were cited as intervening conditions.

Corroborating the findings of Category 2, studies show caregivers’ burden in HC, who are responsible for performing or assisting in daily care, highlighting food, hygiene, administration of medications, monitoring vital signs, dressings, among others, with no time left to take care of themselves or to do other activities of self-interest^(32-33)^.

In Category 3, seven propositions related to strategies for dealing with, living with, intervening in home PC, such as being present and providing love, having faith, and/or a religion. There is evidence that the spiritual need stands out in the face of the final moments of life, because this becomes a therapeutic and humanistic strategy that can reflect on the way in which a person will experience the dying and death process, and this should be considered in the care provided^(34)^. In addition to these, in this research, actions to obtain materials necessary for dressings, hygiene or others, according to a family member’s care needs, were pointed out as a concern, recognizing that the provision by the HCS relieves care management by the family.

In Category 4, 12 contextual conditions (macro and micro) characterized PC at home, managed by the family, clarifying that the family takes over a large part of the costs generated by HC, especially with materials and health services not obtained by SUS. This tightens the family budget even more, as wages are low and spending is high. Sometimes it is necessary to make “sacrifices” to meet, in the best possible way, a family member’s care needs. Talking about the death/dying process experienced by a family member under home PC is a difficulty for caregivers, and family and healthcare professionals. It is worth mentioning that, in the context of care in HC, a significant portion of caregivers is forced to reduce their working hours or even give up their jobs and, consequently, have a reduced source of income, which impacts the family budget^(33)^.

In Category 5, eight intervening conditions stood out, i.e., those that influence or interfere with caregivers’ home PC management. Considering the dialogue with HCS, communication between professionals and caregivers proved to be a relevant condition to be improved, considering the difficulties mentioned to address PC and issues related to the dying and death process. Given this scenario, it is noteworthy that professionals who work with PC often experience pain, death and grief. Thus, technical and interpersonal skills are required, such as empathy, reception and dialogue, to deal with the family in the death and dying process of a person under PC^(35)^.

An important finding of this validation process was that we did not obtain a consensus on which multidisciplinary team professionals “talk openly with family members about the death/dying process”, how they approach the subject, alerting to the need for training, prior preparation and research on communication and language to be adopted with the family within the home PC. Thus, we corroborate with other researchers on the recommendation that the results of PC in home interventions are widely researched, in order to obtain a consensus on this modality^(36)^.

The theoretical matrix validated by CC represented the participants’ reality, providing credibility, consistency and coherence, supported by the research findings, explaining and representing in properties and dimensions^(29)^ home PC management by the family. Its reliability and validity refers to the specific context, presenting, however, a level of abstraction sufficient to its application to different scenarios (HCS)^(12)^. The pedagogical proposal adopted fostered dialogue and (re)signification of the meanings and knowledge of those involved, going beyond the initial role of favoring the theoretical matrix validation stage, as it proved to be a way of educating and promoting the exercise of citizenship by participants in the context of HC^(24-25)^.

The validation process recommends that, in the context studied, caregivers need care, support, guidance by the responsible team, to learn care, according to patients’ needs. Elucidations on how they can contribute to basic care for a family member enable empowerment so that they actively act in the care more satisfied and suffer less from the effect of dealing with the proximity of a family member’s death^(31-32)^.

### Study limitations

A limitation to be considered was the non-application of substantive theory to a new context to reach the control criterion within the scope of the same study, due to the time for its conclusion, which requires a new methodological undertaking and efforts in other studies. It is understood that the application aims to guide subsequent actions, direct possible interventions to be assessed, reinforcing the relevance of new researches that analyze the theory according to this criterion in the context of PC offered through HC.

### Contributions to nursing, health, and public policies

This article awakens to the need for permanent education of nursing and HC multidisciplinary team professionals on communication and language to be adopted with patients and families who live the death/dying process. It instigates discussions about the forms of support to home caregivers, in order to support public policies to support family management in the context of PC offered through HC. Furthermore, it contributes to qualitative research that adopts GT, especially regarding the assessment and validation of substantive or formal theories by researchers in nursing and other areas of health interested in validation studies. CC enable the return of research findings to society, promoting rapprochement between researchers, nurses, other professionals and health service users.

## FINAL CONSIDERATIONS

In this study, we present the GT validation process on home PC management by a caregiver of a family member who experiences the death/dying process. To this end, we used CC as a methodological strategy, which proved to be efficient, because, in addition to validating the theory according to the GT criteria, it allowed for interaction between participants and researchers, the sharing of experiences between the validators and, at the same time, the feedback of research findings to participants and HCS.

Although the explanations generated by the validated theoretical matrix reach relevance to the phenomenon and demonstrate transfer and applicability potential, it is important to emphasize that it does not intend to express the totality in the reality experienced by participants. The matrix integrates, however, meanings and makes sense to it.

We recommend that CC be considered as a possibility for validation of substantive theories resulting from GT and that new experiences developed with this strategy be published, so that it is improved and ratified within the scope of the methodological stage of validating the knowledge produced qualitatively.

By demonstrating the validation process, we aim to sensitize qualitative researchers, especially those who do not master the method in question, so that they understand and replicate this strategy, providing quality and validity in future studies that adopt GT as a method or that are in the process of being finalized.

## References

[1] Ministério da Saúde (BR) (2016). Redefine a Atenção Domiciliar no âmbito do Sistema Único de Saúde (SUS) e atualiza as equipes habilitadas [Internet].

[2] Vasconcelos GB, Pereira PM. (2018). Cuidados paliativos em atenção domiciliar: uma revisão bibliográfica. Rev Adm Saúde.

[3] Matos MR, Muniz RM, Barboza MCN, Viegas AC, Rockembach JA, Lindemann LG. (2017). Representações sociais do processo de adoecimento dos pacientes oncológicos em cuidados paliativos no domicílio. Rev Enferm UFSM.

[4] Silva AE, Braga PP, Sena RR, Duarte ED, Sena LR. (2019). Home palliative care: integrative review. Cienc Cuid Saúde.

[5] Couto AM, Caldas CP, Castro EAB. (2018). Family caregiver of older adults and Cultural Care in nursing care. Rev Bras Enferm.

[6] Gomes B, Calanzani N, Curiale V, McCrone P, Higginson IJ. (2013). Effectiveness and cost-effectiveness of home palliative care services for adults with advanced illness and their caregivers. Cochrane Database System Rev.

[7] Silva GWS, Enders BC, Sousa FGM, Sena JF, Santos RC, Silva AB (2018). Grounded theory in theses and dissertations of brazilian nursing. Texto Contexto Enferm.

[8] Santos JLG, Erdmann AL, Sousa FGM, Lanzoni GMM, Melo ALSF, Leite JL (2016). Methodological perspectives in the use of grounded theory in nursing and health research. Esc Anna Nery.

[9] Andrews T, Mariano GJS, Santos JLG, Koerber-Timmons K, Silva FH (2017). The Methodology of Classic Grounded Theory: considerations on its application in nursing research. Texto Contexto Enferm.

[10] Peiter CC, Santos JLG, Kahl C, Copelli FHS, Cunha KS, Lacerda MR (2020). Grounded Theory: use in scientific articles published in brazilian nursing journals with Qualis A classification. Texto Contexto Enferm.

[11] Santos JLG, Cunha KS, Adamy EK, Backes MTS, Leite JL, Sousa FGM (2018). Data analysis: comparison between the diferentt methodological perspectives of the Grounded Theory. Rev Esc Enferm USP.

[12] Strauss A, Corbin J (2008). Pesquisa qualitativa: técnicas e procedimentos para o desenvolvimento de teoria fundamentada.

[13] Lacerda MR, Nascimento JD, Gomes IM, Hermann AP (2019). Theoretical construction in the Grounded Theory. CIAIQ[Internet].

[14] Adamy EK, Zocche DAA, Vendruscolo C, Santos JLG, Almeida MA (2018). Validation in grounded theory: conversation circles as a methodological strategy. Rev Bras Enferm.

[15] Souza SS, Silva DMGV (2011). Validation of a theoretical model: knowing the interactive processes within the support network for people with tuberculosis. Acta Paul Enferm.

[16] Pio D, Bocchi S, Chirelli M, Vernasque J (2017). Validação de modelo teórico: conhecendo o processo experencial de professores médicos a partir da Teoria Fundamentada nos Dados. CIAIQ [Internet].

[17] Cecílio LCO (2011). Apontamentos teórico-conceituais sobre processos avaliativos considerando as múltiplas dimensões da gestão do cuidado em saúde. Interface Comun Saúde, Educ.

[18] Clarke AE (2003). Situational Analyses: Grounded Theory mapping after the postmodern turn. Symbolic Interaction.

[19] Johnson JL, Adkins D, Chauvin S (2020). A review of the quality indicators of rigor in qualitative research. Am J Pharm Educ.

[20] Torlig EGS, Resende PCR (2019). Validação de Instrumento de Coleta de Dados: Experiência com o Coeficiente de Validação de Conteúdo (CVC) e Proposição de uma Nova Abordagem para Pesquisas Qualitativas. CIAIQ [Internet].

[21] Roman DJ, Osinski M, Erdmann RH (2017). The construction process of grounded theory in administration. Contad Adm.

[22] Leung L (2015). Validity, reliability, and generalizability in qualitative research. J Family Med Prim Care.

[23] Cleland JA (2017). The qualitative orientation in medical education research. Korean J Med Educ.

[24] Dias ESM, Rodrigues ILA, Miranda HR, Corrêa JA (2018). Conversation wheel as education strategy in health for nursing. Rev.

[25] Freire P (2002). Educação como prática da liberdade.

[26] Rieger KL (2019). Discriminating among grounded theory approaches. Nurs Inq.

[27] Girardon-Perlini NMO, Simon BS, Lacerda MR (2020). Grounded Theory methodological aspects in Brazilian nursing thesis. Rev Bras Enferm.

[28] Moura ABF, Lima MGSB (2014). A reinvenção da roda: roda de conversa, um instrumento metodológico possível. Interfaces da Educ [Internet].

[29] Melo MCH, Cruz GC (2014). Roda de Conversa: uma proposta metodológica para a construção de um espaço de diálogo no Ensino Médio. Imagens Educ.

[30] Velloso ISC, Tizzoni JS (2020). Critérios e estratégias de qualidade e rigor na pesquisa qualitativa. Cien Enferm.

[31] Silva YC, Silva KL (2020). Constituição do sujeito cuidador na atenção domiciliar: dimensões psicoafetiva, cognitiva e moral. Esc Anna Nery.

[32] Silva YC, Silva KL, Velloso ISC (2021). Practices used by a home care team: implications for caregivers. Rev Bras Enferm.

[33] Delalibera M, Barbosa A, Leal I (2018). Circunstâncias e consequências do cuidar: caracterização do cuidador familiar em cuidados paliativos. Ciên Saúde Coletiva.

[34] Monteiro T, Amorim T, Porto A, Carbogim F, Ferreira M, Thofehrn M (2021). Constructing the meaning, to an oncological nursing team, of spirituality in the process of dying. Rev Enferm UERJ.

[35] Nardino F, Olesiak LR, Quintana AM Significações dos cuidados paliativos para profissionais de um serviço de atenção domiciliar. Psicol: Ciên Prof.

[36] Valadares GV, Hang AT, Santana GO, Santos GLA, Rosa LS, Santos SS, Lacerda MR, Santos JLG (2019). Teoria Fundamentada nos Dados: bases teóricas e metodológicas.

